# Prevalence of herpesvirus, polyomavirus, and respiratory viruses in saliva from a Quilombola community in the Brazilian Amazon

**DOI:** 10.1007/s42770-026-02110-8

**Published:** 2026-10-12

**Authors:** Wanderley Pansani, Samuel Nascimento Santos, Érica Correia Ramos, Barbara Júlia de Mello, Jane Glébia de Abreu, Matheus Esteves Fernandes, Pablo Fortunato da Silva, Guilherme José Costa Silva, Jane de Eston Armond, Stella de Souza Vieira, Jonatas Bussador do Amaral, André Luis Lacerda Bachi, Luiz Henrique da Silva Nali

**Affiliations:** 1 Post-graduation Program in Health Sciences, Santo Amaro University, São Paulo, Brazil; 2 School of Dentistry, Santo Amaro University, São Paulo, Brazil; 3 Ent Research Lab, Department of Otorhinolaryngology -Head and Neck Surgery, Federal University of Sao Paulo, São Paulo, Brazil; 4 Post-graduation Program in One Health, Santo Amaro University, São Paulo, Brazil

**Keywords:** Polyomaviridae, Herpesviridae, Respiratory viruses, Inflammatory cytokines, Quilombola community

## Abstract

**Introduction:**

Quilombola communities, composed of descendants of enslaved individuals from Brazil’s colonial period, often face limited access to healthcare, which may increase their susceptibility to viral infections. Oral transmission routes, particularly through saliva, are relevant for viruses such as those in the Polyomaviridae family. Therefore, here we aimed to investigate the prevalence of HSV-1, JC (JCV), and BK polyomaviruses, and respiratory viruses (Influenza A/B, RSV, SARS-CoV-2), as well as the salivary inflammatory profile in individuals from a Quilombola community.

**Methods:**

A cross-sectional study was conducted in the Quilombola community of Mangabeira, Pará, Brazil. Unstimulated saliva samples were collected from 100 individuals. Oral health was assessed, and nucleic acids were analyzed using real-time PCR (JCPyV, BKPyV, HSV-1) and multiplex TaqMan PCR (RSV, Influenza A/B, SARS-CoV-2). Cytokines (IL-6, IFN-γ, TNF-α, IL-10) were quantified.

**Results:**

BKPyV was detected in 19%, while HSV-1 and SARS-CoV-2 were found in 3% each. No cases of JCPyV, RSV, or Influenza were detected. BKPyV infection was associated with elevated IFN-γ (*p* < 0.01). SARS-CoV-2 and HSV-1 infections were linked to pro-inflammatory cytokine profiles. No association was found between viral detection and tooth loss or the use of prosthesis.

**Conclusion:**

A high prevalence of BKPyV and a salivary pro-inflammatory response were observed, highlighting potential oral health vulnerabilities in this underserved population.

**Supplementary Information:**

The online version contains supplementary material available at https://doi.org/10.1007/s42770-026-02110-8.

## Introduction

Quilombola communities are self-sustaining groups that organize themselves to ensure their own subsistence. They are mainly composed of individuals of Afro-descendant ancestry, descended from those who resisted slavery during Brazil’s colonial period [1]. According to the 2022 national demographic data (Brazilian CENSO), these communities comprise approximately 1.32 million individuals (0.65% of the Brazilian population), living in nearly 474,000 households across nearly 1700 municipalities in Brazil. The Northeast region is home to about 68.15% of these residences [2]. In addition, less than 6% of the Quilombola population lives in demarcated areas, which enhances the reality faced by them, marked by systemic discrimination and socioeconomic exclusion. In fact, Quilombola people have limited access to healthcare services, primarily due to long waits and geographical barriers, as well as food insecurity, which underscores the social and health vulnerabilities of these populations.

Considering this condition, it is feasible to suppose that there is a lack of information regarding the oral health and possible transmission of diseases, especially by viruses. For instance, there are a few studies that focused on understanding the prevalence of HTLV-1/2 [3], Papillomaviruses [4], Norovirus [5], and SARS-CoV2 [6] in these populations, and only a few others have assessed their oral health conditions [7]. Despite the known circulation of several viruses in vulnerable Brazilian populations, no studies have investigated the salivary shedding of BK Polyomavirus(BKPyV), JC Polyomavirus(JCPyV), Herpes simplex virus (HSV-1), and respiratory viruses in Quilombola communities. Therefore, the aim of this study was to assess not only the prevalence of JCPyV, BKVPyV, HSV-1, Influenza A, Influenza B, Respiratory Sincicial Virus (RSV), and Severe Acute Respiratory Syndrome Coronavirus 2 (SARS-CoV-2) infections but also its association with inflammatory status and oral health, through saliva, in individuals residing in the Quilombola community of Mangabeira, located in the Amazon region of Pará state.

## Methods

### Study population and ethical considerations

This is a cross-sectional observational study that was conducted in Mangabeira Village, a Quilombola community situated within the Amazon rainforest on the banks of the Tocantins River (Coordinates 2°36′43″ S; 49°37′13″ W). This major river separates the community from the municipal center of Mocajuba, located in the state of Pará. Municipality is characterized by low socioeconomic indicators, as reflected by a Human Development Index (HDI) of 0.575 [8].

A total of 100 volunteers were included in the study during May to July 2024, which represents around 12,5% of the village population. The study population consisted of both men and women aged 19 to 79 years, and they were selected randomly. Anthropometric data were collected, and the volunteers’ oral health (including the number of teeth and use of dental prostheses) was assessed by a dentist. Inclusion criteria were: being 18 years of age or older, residing in the village, and voluntarily agreeing to participate by signing the informed consent form. Exclusion criteria included refusal to participate, inability to provide a saliva sample, or incomplete clinical and demographic information. Volunteers were stratified into two groups according to the World Health Organization Functional Dentition (WHO-FD) [9] criteria as follows: Group 1, composed by those with more than 20 teeth and Group 2, composed by those who presented less than 20 teeth This study was approved by the ethical committee of the Universidade Santo Amaro under project #72816823.7.0000.0081. Volunteers were included in the study after signing the written consent form. Importantly, this study was carried out in agreement with the Declaration of Helsinki and ethical guidelines outlined in Resolutions 466/2012 and 510/2016 of the Brazilian National Health Council. Also, this study was registered on the *Sistema Nacional de Gestão do Patrimônio Genético e do Conhecimento Tradicional Associado (SISGen) under protocol #A7CC91D.*

### Saliva samples collection and preparation

Unstimulated whole saliva samples were self-collected by the volunteers. In this respect, the volunteers were oriented to accumulate the saliva on the floor of the mouth and, after, letting it passively flow from the lip directly into 15 mL sterile conical tubes (Sarstedt, Nuremberg, Germany). Samples were labeled with alphanumeric codes in order to preserve the identity of the study participants. After collecting, the samples were kept on ice during transport until being stored frozen at −20^o^ C. The samples were transported in ice dry until delivered to the research laboratory at Universidade Santo Amaro. Subsequently, the saliva samples were centrifuged at 1500 g for 10 min. The resulting supernatant was used to determine the salivary inflammatory status, and the cell pellet was resuspended in 200 µL in sterile PBS and sent for molecular analyses for virus detection.

### Determination of salivary cytokine concentration

To determine the salivary concentrations of the cytokines IL-6, IFN-γ, TNF-α, and IL-10, all samples were analyzed using enzyme-linked immunosorbent assay (ELISA). The procedure was carried out with commercial kits from Thermo Fisher Scientific (Waltham, Massachusetts, USA), specific to each analyte, following the manufacturer’s instructions. Detection was performed at a wavelength of 450 nm, and the absorbance values were used to determine the concentration of each analyte (pg/mL) based on the standard curve provided by the kit.

### Viral detection

Nucleic acid for viral detection was obtained using the PureLink™ Viral RNA/DNA Mini Kit Thermo Fisher Scientific (Waltham, Massachusetts, USA) and was stored in −80^o^ C until use. Prior to RNA virus-specific assays, and aliquot was used to verify the quality of the extracted material by DNAse treatment with DNA-free nuclease Ambion – ThermoFisher (Waltham, Massachusetts, USA), cDNA reconstruction followed by real-time PCR using primers targeting the endogenous human GAPDH gene [10]. The reaction mix included 1× SYBR Green Master Mix, 3.2 pmol of each primer, and nuclease-free H₂O to a final volume of 25 µL. The cycling conditions were as follows: initial denaturation at 95° C for 10 min, followed by 40 cycles of 95° C for denaturation and 60° C for primer annealing and extension. A final melting curve analysis was performed to distinguish positive from negative samples. Another aliquot without DNAse treatment was used to for testing the DNA-specific viruses in each sample. The primers used for HSV-1 [11], JCV [12, 13], and BK virus [13, 14] were based on previously published sequences and the cycling conditions were the same for GAPDH gene [10]. Finally, to detect the respiratory viruses, which included Influenza A, Influenza B, RSV, and SARS-CoV-2, we used the Allplex™ SARS-CoV-2/FluA/FluB/RSV assay Seegene (Seoul, South Korea). The cycling conditions for this assay consisted of incubation at 37 °C for 30 min, followed by 40 cycles of 95° C for 45 s and 60° C for 1 min. Samples were tested in duplicate, and In all reactions positive and negative controls were included.

### Statistical analysis

To compare scores between different groups, both parametric and non-parametric tests were applied depending on the normality of data distribution. For group comparisons we performed using either the T-Student test or Mann–Whitney test, as appropriate. All data were entered into a database created using GraphPad Prism version 9.0 (La Jolla, California, USA) and analyzed using descriptive statistics, including percentage calculations, frequencies, and means, as well as graphical representations.

## Results

The demographic findings and the oral health status of the volunteers are summarized on Table 1.

**Table 1:** Sociodemographic and oral health data of the study participants

	*Men (n = 40)*	*Women (n= 60)*	*Total(n = 100)*
Age range (avg/SD)	20–79(33.2/17.7)	19–78(34.01/18.5)	19–79(33.6/19.43)
*Weight range (SD)*	40–97,8(13.4)	35–80,4(12.1)	33–97(13.1)
*Height cm (SD)*	135–177(9.4)	153–185(7.1)	140–185(9.8)
*BMI avg (SD)*	25.9(3.8)	25.1(3.6)	25.4(3.7)
*> 20 teeth n = 79 (SD)*	24–32(2.9)	22–32(2.7)	22–32(2.8)
*< 20 teeth n=21 (SD)*	3–20(5.6)	7–20(4.2)	3–20(5.9)
*Uses dental prosthesis (%)*	6(6)	9(9)	15(15)
*Does not use dental prosthesis (%)*	34(34)	51(51)	85(85)

Legends: *AVG*= average,* SD*= Standard Deviation,* BMI *= Body Mass Index [2]

### Detection of viral DNA/RNA in salivary samples

Overall, BKPyV DNA was detected in 19 out of 100 samples (19%), HSV-1 DNA in 3 out of 100 samples (3%), and SARS-CoV-2 RNA in 3 out of 100 samples (3%). No JCV DNA or RNA from Influenza A, Influenza B, or RSV was detected in any of the tested samples.

Only 1 out of the 19 individuals who tested positive for BKPyV was a user of a dental prosthesis. Additionally, as shown in Table 2, two volunteers positive for BKPyV presented co-infections with other viruses. It is also noteworthy that all volunteers who tested positive for BKPyV had more than 20 natural teeth present.

**Table 2:** Frequency of HSV-1, BKPyV, and SARS-CoV-2 detection in study participants and associations with sociodemographic, anthropometric, and oral health variables

	BKVPyV+(*n* = 19)	HSV1+(*n* = 3)	SARS-CoV2(*n* = 3)
Sex(%) Men(40) Women(60)	9(22,5) 10(16,7)	1(2,5) 2(3,4)	1(2,5) 2(3,4)
Age(n) *17–30(51)* *31–40(12)* *41–60(24)* *60+ (13)*	14 4 0 1	2 0 0 1	1 0 0 1
BMI(n) *19–24(47)* *25–29(40)* *30–34(9)* *35–39(4)*	10 6 3 0	2 1 0 0	0 2 1 0
Oral health *< 20 teeth* *> 20 teeth*	0 19(100)	0 3(100)	0 3(100)
*Simultaneous viral shedding*	1(HSV-1) 1(SARS-CoV2)	1(BKVPyV)	1(BKVPyV)

Legend: *BMI *= Body Mass Index. No statistically significant differences were observed among the analyzed variables

### Inflammatory profile in the saliva of study participants

**Fig. 1:**
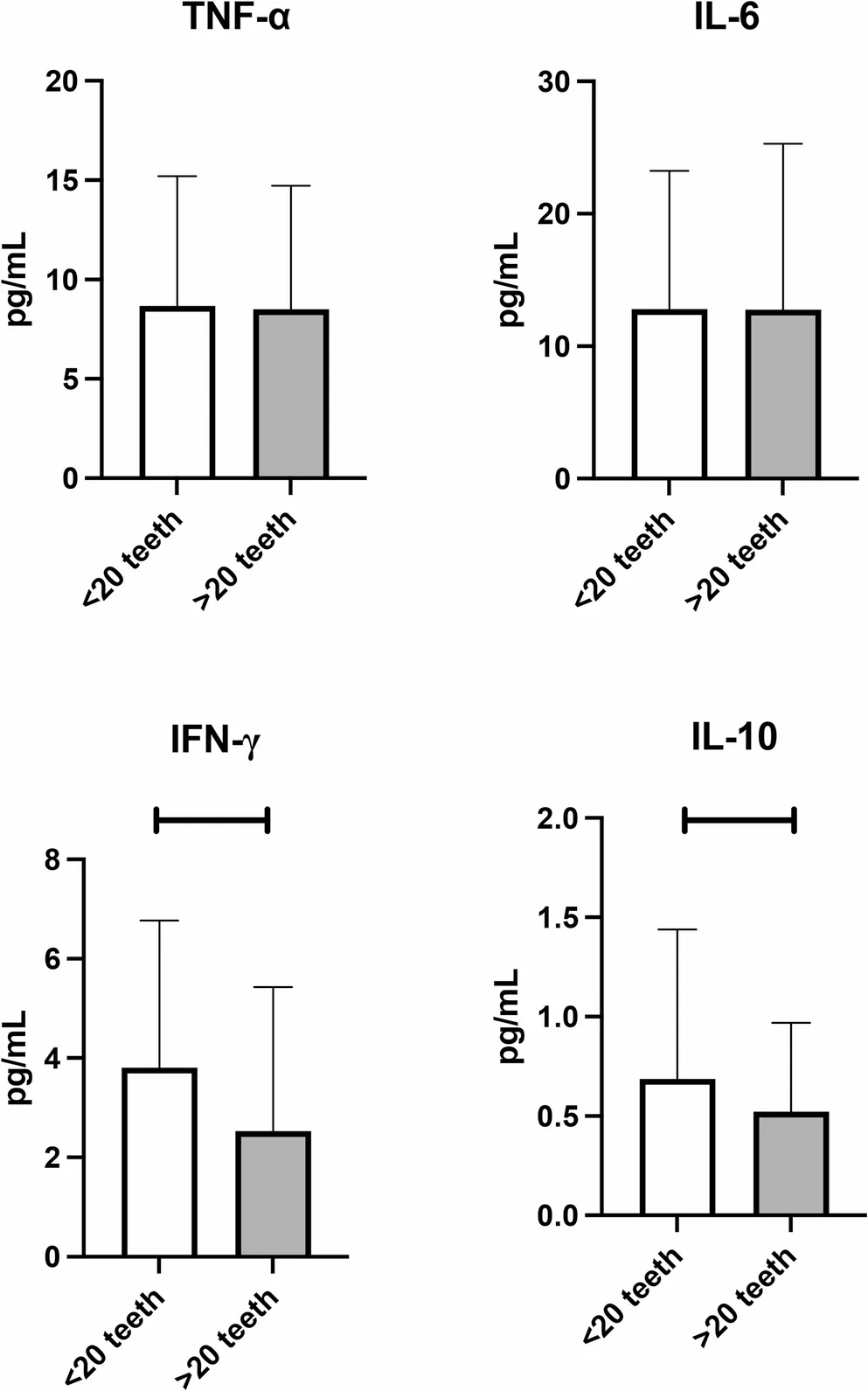
Overall analysis of pro- and anti-inflammatory cytokine concentrations in the saliva of study participants. Data are expressed in median and confidence interval. Mann-Whitney test Legend: *=*p* < 0.0,1 *n* < 20teeth = 21 and *n*>20teeth = 79

Figure 1 highlights the salivary concentrations of the pro-inflammatory cytokines TNF-α, IFN-γ, IL-6, and the anti-inflammatory cytokine IL-10. It can be observed that the volunteers with fewer than 20 teeth exhibited significantly higher levels of IL-10 and IFN-γ.

**Fig. 2:**
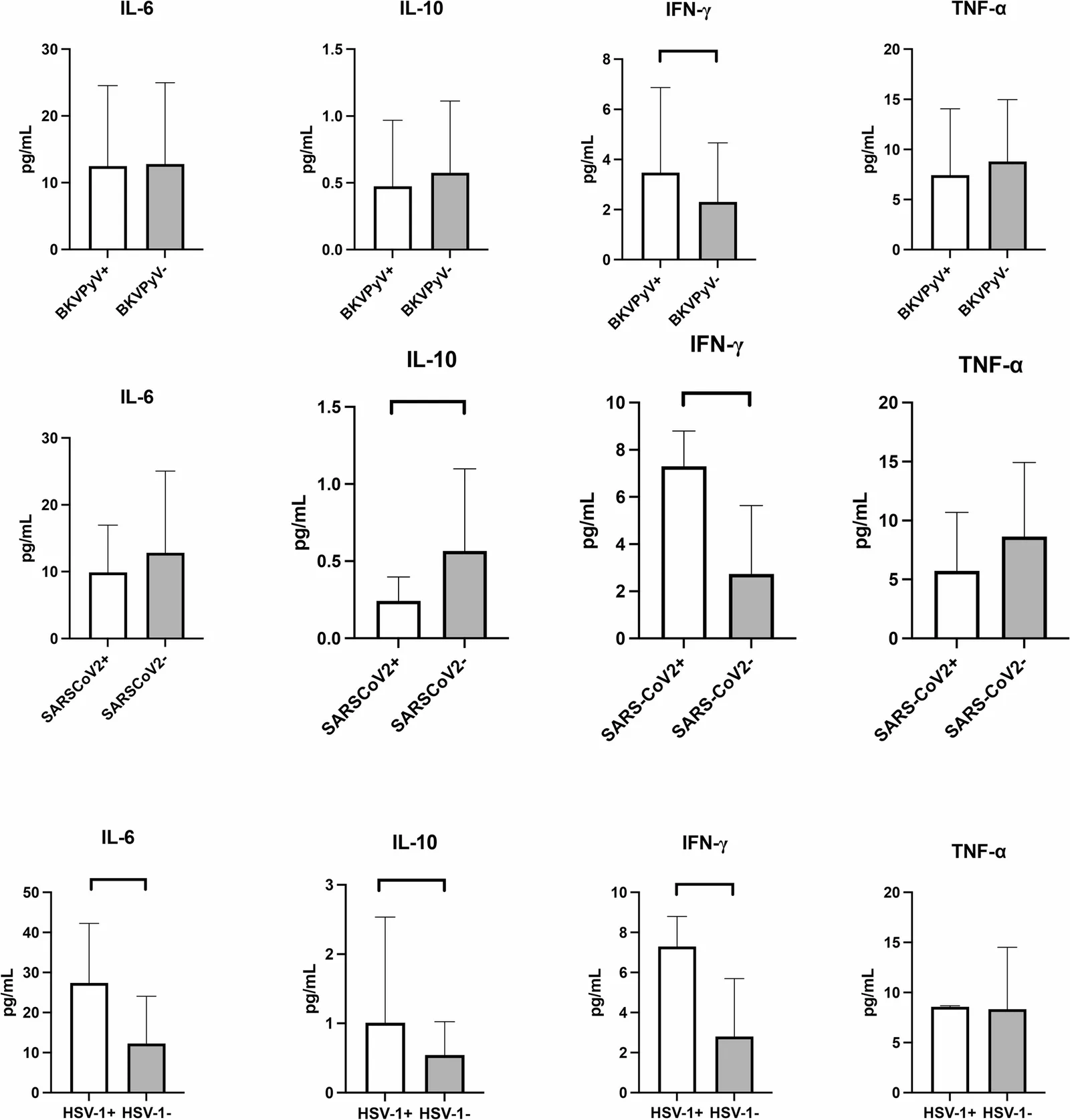
Cytokine salivary concentrations according to BKPyV, SARS-CoV2 and HSV-1 positivity status. Data are expressed in median and confidence interval. Mann-Whitney test Legend: *=*p* < 0.01

Subsequently, we analyzed the frequency of BKPyV, SARS-CoV-2, and HSV-1 detection in relation to salivary levels of pro- and anti-inflammatory cytokines. Volunteers who tested positive for BKPyV exhibited higher salivary levels of IFN-γ (*p* < 0.01), and those who tested positive for SARS-CoV-2 exhibited higher levels of IFN-γ and lower levels of IL-10 (*p* < 0.01). Finally, we analyzed the cytokine concentrations in the volunteers who were infected and excreting HSV-1 in their saliva. The data revealed that these individuals exhibited higher levels of IL-6, IL-10, and IFN-γ compared to those who were not infected with HSV-1 (*p* < 0.01). All these findings can be observed in Fig. 2. Also, we have compared the cytokine salivary concentrations between the individuals who presented co-infection compared to those who presented single viral infection and no statistical relevance was observed.

## Discussion

The main finding presented here provides potentially novel data, as there are no previous studies describing the prevalence of BKPyV in Quilombola populations. This virus is known for its persistent infection and can be excreted in the urine and saliva of immunocompetent individuals [15], however BKPyV is associated with urinary tract diseases, particularly in individuals with immunosuppressive conditions, such as transplant recipients or those infected with HIV [16]. Although more commonly observed in immunocompromised individuals, BKPyV, as a member of the Polyomaviridae family, may present a carcinogenic role [17]. Indeed, this infection is often asymptomatic in immunocompetent individuals, which likely favors prolonged infection. Furthermore, it is estimated that polyomaviruses infect humans during childhood or adolescence. Importantly, BKPyV does not exhibit replication exclusively in the urinary tract and other body fluids remain underexplored. However, we are in line with previous studies that have reported the presence of viral DNA in saliva samples from both immunocompromised and healthy individuals [18], which may represent that salivary glands cells are indeed a site for BKVPyV tropism rather than residual DNA from infection in other sites, since previous studies demonstrated the infection of salivary gland cells by BKVPyV in vitro [19]. All of these findings underscore the importance of saliva as a route for BKVPyV shedding, suggesting the active and possibly persistent replication, which could certainly serve as an alert for head and neck tumors. In line with this observation, a systematic review with meta-analysis described that individuals infected with BKPyV have a higher risk of developing oral cancer compared to uninfected individuals [20]. In addition to this finding, the fact that all of BKPyV positive individuals had had more than 20 natural teeth might reveal few scenarios where it may reflect the persistence of a lifelong latent infection in immunocompetent individuals hosts, as BKPyV commonly remains asymptomatic and controlled by the immune system in immunocompetent individuals [21] and also may not reflect a condition of disease and risk of tooth loss. The maintenance of a functional dentition may also reflect a lower cumulative burden of oral diseases. Previous studies have shown that more extensive periodontal disease is associated with increased circulating levels of inflammatory mediators, including C-reactive protein, IL-6, and TNF-α [22, 23]. In this context, the presence of at least 20 natural teeth may indicate a more favorable oral health history and a lower cumulative inflammatory burden, although the number of teeth alone does not provide a direct assessment of systemic or immune status.

Importantly, the volunteers live in a region where the HDI is 0.57 [2], which is considered to be low according to the United Nations Development Programme (UNDP). In addition, the per capita income of the municipality is not available on the social development indicators from the municipal government. This scenario reveals an even more concerning when considering the people that we have investigated, a population composed of historically socially vulnerable individuals, which raises additional concerns about the development of clinical manifestations related to this or other viral infections Furthermore, it is important to note that there is no antiviral therapy available for the treatment of BKPyV, highlighting the fact that BKPyV infection, especially in immunocompetent individuals, is a phenomenon that tends to be neglected.

Less prevalently, we also identified a 3% (3/100) prevalence of HSV-1 infection. Few studies have investigated the prevalence of HSV-1 infection in vulnerable populations such as Quilombola communities as follows: some studies have examined the presence and seroprevalence of HSV-1 in Indigenous populations, for instance, the study conducted by Bonfim et al. which reported IgG seroprevalence of 97% and IgM of 20%, as well as the presence of HSV-1 DNA in plasma samples showeda prevalence of 25.6% [24]. While serological positivity reflects previous exposure and lifelong viral persistence, salivary molecular detection depends on active viral shedding, which occurs intermittently and may not be present at the time of sample collection. Therefore, further in-depth studies focusing on other Quilombola populations are warranted to determine the dissemination of HSV-1 in vulnerable communities. Additionally, the detection of HSV-1 in saliva samples tends to show low prevalence, even among immunocompromised individuals [25].

SARS-CoV-2 was detected in 3% of the study participants. Unlike HSV-1, this prevalence demonstrate that viral circulation may still be occurring in the community, despite the end of the SARS-CoV2 global public health emergency. This may be concerning and raises potential issues regarding the vaccination status of the participants. A recent study reported that the prevalence of SARS-CoV-2 infection tends to be higher in Quilombola communities compared to general population [6]. This may help explain the prevalence observed in our study population, even after the end of the global emergency period caused by the SARS-CoV-2 pandemic. In fact, during the pandemic period of SARS-CoV2 in Brazil, a previous study has described that Quilombola population presented low frequency of anti-SARS-CoV2 IgG, which revealed that although isolated, they could be potentially at risk [26]. In addition, a more recent study revealed concerning finding regarding the SARS-CoV2 vaccine coverage, which was around 48.43% for the first dose and 49,41% for the second dose [27].All together, these findings may reflect barriers to accessing healthcare services and immunization. Undoubtedly, even in the post-emergency context, prevention strategies must be implemented, particularly in populations living in conditions of vulnerability.

We also compared cytokine profiles between individuals infected with BKVPyV, HSV-1, and SARS-CoV-2 and those who were not infected. In all comparisons, we found statistically significant differences in at least one cytokine. In BKVPyV-infected individuals, IFN-γ levels were higher, consistent with their role in antiviral control. Previous research demonstrated that IFN-γ can reduce early T antigen expression, interfering in the BKVPyV replication [28]. Additionally, the intermittent shedding pattern of BKVPyV, which was previously described [29] may reflect the viral persistence condition might be dependent on host immune activation, particularly IFN-mediated responses, indicating that changes in the production of IFN-γ might be regulate the viral replication in the saliva as well. HSV-1-infected individuals had elevated IL-6, IL-10, and IFN-γ levels, indicating a robust inflammatory response alongside regulatory mechanisms. Notably, IL-6 plays a role in HSV-1 latency, as it can promote transcription factors that enhance viral replication [30]. Interestingly, HSV-1 can later downregulate IL-6 as an immune evasion strategy [31], highlighting the dynamic regulation of this cytokine during infection. Also, IFN-γ is responsible for controlling the viral replication after in the lytic infection, more specifically interfering in the correct assembly of microtubules, necessary for viral transport and transmission between cells [32], while IL-10 expression induces a local protective anti-inflammatory condition during HSV-1 infection, usually regulated by the viral infection that leads to suppressor of cytokine signaling-3 (SOCS-3) production [33], favoring the viral shedding.

SARS-CoV-2-infected individuals also showed increased IL-10 and IFN-γ levels, consistent with the virus’s known ability to trigger strong inflammatory and antiviral responses. A recent meta-analysis confirmed elevated systemic IFN-γ levels during the acute phase of COVID-19 [34], underscoring the pro-inflammatory nature of SARS-CoV-2 infection. On the other hand, IL-10 increased serum levels are described in patients infected by SARS-CoV2 [35], which is in line with our findings, indicating that both systemic profiles of high concentration of IL-10 are found in the serum but also as in the saliva. Importantly, the high concentration of IL-10 is believed to be crucial in order to prevent severe cases of COVID-19 and prevent neurological manifestations usually associated with the high pro-inflammatory profile of the disease.

Finally, the differences observed according to dentition status may reflect distinct immunological profiles associated with cumulative biological exposures throughout life. Tooth loss has been recognized as a marker of long-term oral health status and has been associated with aging-related conditions and chronic low-grade inflammation [36, 37]. In this context, the higher levels of IFN-γ observed among individuals with fewer remaining teeth may suggest a more pronounced pro-inflammatory immune environment, whereas alterations in IL-10 levels may reflect differences in regulatory mechanisms involved in maintaining immune homeostasis. Together, these findings support the hypothesis that dentition status may be associated with variations in the balance between inflammatory and anti-inflammatory responses, potentially influencing susceptibility to persistent viral infections and other age-related immune processes.

Importantly, our study present some limitation that should be addressed: (i) our study have only investigated a portion of the community since we have recruited the volunteers by conveniency, and certainly further studies should be performed in with higher n in order to better understand the prevalence of these viral infection in quilombola communities; (ii) we have investigated for the frequency of some vaccine preventable viral infections such as Influenza and SARS-CoV2, but due to the lack of information the vaccine history was not available which could potentially impact the real immunological conditions against these viral diseases. However, as mentioned, previous SARS-CoV2 vaccine coverage is bellow than ideal, so our findings may still reveal a concerning scenario and; (iii) Our study revealed a relatively low number of participants positive for some of the investigated viruses. As a result, comparisons of cytokine profiles between virus-positive and virus-negative individuals may have been underpowered, however even though the low positive participants the comparison revealed statistical significancy between the comparison, which might indicate the possible associations observed.

## Conclusion

In summary, this study reveals a high prevalence of BKPyV and detectable SARS-CoV-2 shedding in saliva from a Quilombola population, highlighting potential gaps in surveillance and vaccination coverage. These findings provide potentially novel insights, given the scarcity of studies investigating the presence of these viruses in the saliva in socially vulnerable communities. Importantly, our results raise concerns regarding not only the risk of neglected viral infections, particularly by BKVPyV, due to its typically subclinical presentation, but also its potential oncogenic nature that may pose an increased risk for head and neck cancer development. In addition, the observed SARS-CoV-2 prevalence reinforces the importance of targeted public health strategies for vulnerable groups. Lastly, our data also shed light on the mucosal inflammatory responses associated with infection by different viruses.

## Supplementary Information

Below is the link to the electronic supplementary material.

Supplementary Material 1 (DOCX 13.2 KB)

Supplementary Material 2 (PDF 69.1 KB)

## Data Availability

The datasets generated during and/or analysed during the current study are not publicly available due to local ethical legislation that requires that personal information should not be public available but are available from the corresponding author on reasonable request.
