# Supplementary material for: Prevalence of herpesvirus, polyomavirus, and respiratory viruses in saliva from a Quilombola community in the Brazilian Amazon
Source: Braz J Microbiol. 2026 Oct 12;57(1):292. doi: 10.1007/s42770-026-02110-8 (PMC13658731; doi:10.1007/s42770-026-02110-8)
Supplement: Supplementary file 1 [file 42770_2026_2110_MOESM2_ESM.pdf]

Ministério do Meio Ambiente
CONSELHO DE GESTÃO DO PATRIMÔNIO GENÉTICO

SISTEMA NACIONAL DE GESTÃO DO PATRIMÔNIO GENÉTICO E DO CONHECIMENTO TRADICIONAL ASSOCIADO

Comprovante de Cadastro de Acesso

Cadastro nº A7CC91D

A atividade de acesso ao Patrimônio Genético, nos termos abaixo resumida, foi cadastrada no SisGen, em atendimento ao previsto na Lei nº 13.123/2015 e seus regulamentos.

Número do cadastro:	**A7CC91D**
Usuário:	**GUILHERME JOSE DA COSTA SILVA**
CPF/CNPJ:	**068.059.716-67**
Objeto do Acesso:	**Patrimônio Genético**
Finalidade do Acesso:	**Pesquisa**

Espécie

Human polyomavirus 1

Human alphaherpesvirus 1

Severe acute respiratory syndrome coronavirus 2

Título da Atividade:	**Prevalence of Herpesvirus, Polyomavirus, and Respiratory Viruses in Saliva from a Quilombola Community in the Brazilian Amazon**

Equipe

**GUILHERME JOSE DA COSTA SILVA**	**Universidade Santo Amaro - UNISA**
**Luiz Henrique da Silva Nali**	**Universidade Santo Amaro - UNISA**
**Wanderley Pansani Junior**	**Universidade Santo Amaro - UNISA**

Resultados Obtidos

Divulgação de resultados em meios científicos ou de comunicação

Identificação do meio onde fo divulgado:	**Artigo científico intitulado "Prevalence of**

Data do Cadastro:	**04/09/2026 14:03:32**
Situação do Cadastro:	**Concluído**

Conselho de Gestão do Patrimônio Genético
Situação cadastral conforme consulta ao SisGen em **14:04** de **04/09/2026**.

SISTEMA NACIONAL DE GESTÃO DO PATRIMÔNIO GENÉTICO E DO CONHECIMENTO TRADICIONAL ASSOCIADO - SISGEN
