# Supplementary material for: Prevalence of herpesvirus, polyomavirus, and respiratory viruses in saliva from a Quilombola community in the Brazilian Amazon
Source: Braz J Microbiol. 2026 Oct 12;57(1):292. doi: 10.1007/s42770-026-02110-8 (PMC13658731; doi:10.1007/s42770-026-02110-8)
Supplement: Supplementary file 2 [file 42770_2026_2110_MOESM1_ESM.docx]

| Oligonucleotides | Forward Primer | Reverse Primer |
| --- | --- | --- |
| HSV-1 | CGGCCGTGTGACACTATCG | CTCGTAAAATGGCCCCTCC |
| JCPyV | ATGTTTGCCAGTGATGATGAAAA | GGAAAGTCTTTAGGGTCTTCTACCTTT |
| BKPyV | GAAACTGAAGACTCTGGACATGGA | GGCTGAAGTATCTGAGACTTGGG |
| GAPDH | ACCCACTCCTCCACCTTTGAC | TGTTGCTGTAGCCAAATTCGTT |

Supplementary table 1. Primer sequences used on the study.
